# Treatment of Ingrowing Toe-Nail by Perchloride of Iron

**Published:** 1861-11

**Authors:** 


*Treatment of Ingrowing Toe-Nail by Perchloride of Iron.*—This painful affection, which has seemed to obstinately resist all treatment for its radical cure short of excision, is said to be permanently relieved by the application of perchloride of iron. The suggestion is from M. Wahn, Physician of the Military Hospital at Nice, and is published in the *Gazette des Hopitande.* M. Wahn gives the following account of his experience in the treatment of his own case :

"I was struck with the idea that, if I could dry up or even *tan* the diseased surface, so that the ulcer might be converted into a firm surface, capable of resisting the cutting action of the edge of the nail, I might obtain a complete cicatrization, and consequently a cure. Running over in my mind the most energetic tanning substances, I decided on employing the *perchlorure de fer.* I obtained some in a powdered form, and insinuated it as deeply as possible between the free edge of the nail and the ulcer. I felt almost immediately a moderate sensation of pain, accompanied by a feeling of constriction and a strong, burning sensation. A quarter of an hour after I attempted to walk, and, to my great satisfaction, I found I could bear my weight on my foot throughout its entire length without the least pain—a thing which I had not done before for many months. The following day I carefully examined the diseased parts, and found them mummified and as hard as wood. I applied a fresh quantity of perchlorure de fer, which I allowed to remain for a quarter of an hour ; but I have reason to believe this application was useless, as the mummification was complete by the first process. I continued to walk without the least thought of *ongle encarne,* and about three weeks after was able, by means of a pediluvium, to remove the hardened layer of skin, under which I found a tissue of new formation, which perfectly resisted the pressure of the edge of the nail. Shortly after the whole had returned to its normal condition, and, since, more than two years have passed without a return of the disorder."—*Boston Medical Journal.*

